# The identity of implant materials governs the antimicrobial efficacy of SET-M33

**DOI:** 10.1038/s41598-025-99808-w

**Published:** 2025-05-10

**Authors:** Alessia Maranesi, Sajad Mohammadi, Ismael Castañon, Felipe Gama-Franceschi, Chiara Falciani, Alessandro Pini, Laura Mezzanotte, Wendy Unger, Aldo Ferrari

**Affiliations:** 1 Department of Material Science and Engineering, Universitat Politècnica de Catalunya, Barcelona, 08019 Spain; 2 Hylomorph AG, Technopark, Zurich, 8005 Switzerland; 3 Department of Pediatrics, Laboratory of Pediatrics, Erasmus MC - Sophia Children’s Hospital, Erasmus University Medical Centre, Rotterdam, The Netherlands; 4 Department of Medical Biotechnologies, University of Siena, Siena, 53100 Italy; 5 Department of Radiology & Nuclear Medicine, Erasmus MC, Erasmus University Medical Centre, Rotterdam, The Netherlands; 6 SetLance srl, Siena, 53100 Italy; 7 Department of Molecular Genetics, Erasmus MC, Erasmus University Medical Centre, Rotterdam, The Netherlands

**Keywords:** Implant materials, Surgical pocket infection, Antimicrobial peptides, Resistant bacteria, Protective envelopes, Biotechnology, Cardiology, Health care, Materials science

## Abstract

The physical and chemical properties of implanted materials play a key role in their interaction with synthetic peptides that exert antimicrobial activity. In this study, we explored the diffusion properties and efficacy of the SET-M33 antimicrobial peptide in combination with artificial substrates, comprising cardiac implantable electronic devices (CIEDs) or porous protective envelopes. We found that porous materials, such as biosynthesized cellulose, polymeric meshes, and electrospun membranes, were conducive to SET-M33 diffusion. The diffusion dynamics was controlled by the intrinsic fibrous architecture of the materials. Biosynthesized cellulose supported the peptide’s antimicrobial activity against *E. coli* and *S. aureus.* The efficacy of SET-M33 was instead reduced when combined with the other tested porous membranes and non-porous CIED interfaces, such as titanium and silicone. On the other hand, the low porosity of biosynthesized cellulose membranes, while effective in retaining the drug, diminished diffusion and thus peptide availability. In light of these findings, the implications for the use of antimicrobial peptides in the prevention of CIED surgical pocket infections are discussed.

**Supplementary Information:**

The online version contains supplementary material available at 10.1038/s41598-025-99808-w.

## Introduction

Implant infections have specific characteristics that make them different from, and more insidious than, local or systemic infections driven by the same agents in the absence of a foreign interface^1^. Contamination of an implant site by opportunistic pathogens, entering the pocket during the deployment of synthetic biomaterials, can lead to the colonization of the implant surface. Despite careful perioperative antiseptic protocols, bacterial infections are relatively frequent and typically more serious when associated with permanent prosthetics, such as cardiovascular devices (e.g. cardiac implantable electronic devices; CIEDs)^2^. Here, surgical pocket contamination requires systemic antibiotic treatment and immediate device extraction. Even when treated promptly, CIED infections tend to recur and become chronic, increasing mortality and healthcare costs^3^. For this reason, electrophysiology has been at the forefront of prevention and treatment of surgical pocket infections connected to permanent implants^4^.

The chemical and physical surface properties of implant materials govern their interaction with the surrounding cells and tissues^5^. Micro- and nano-scale geometries have been exploited to modulate the binding of biological molecules, and the ensuing adhesion of eukaryotic and prokaryotic cells^6^. Surface roughness has an impact on hydrophilicity, which is measured as the water static contact angle, and defines the ability of one surface to interact with soluble molecules^7^. Depending on the arrangement, symmetry, and size of the topographic features the hydrophilicity of a material can be increased or decreased, without interfering with other parameters such as the mechanical properties or the surface charge^8^. This principle can be used to tailor the interaction between an implant material and the biological fluids that will be in contact with it upon deployment in the body^9^. Topographies that modify surface properties to demote interaction with biological molecules, such as proteins and sugars, have the intrinsic effect of reducing biological coating (i.e. fouling)^10^. Anti-fouling surfaces have long been studied as a possible solution to dampen the inflammatory response to biomaterials^11^ and to reduce biofilm formation^12^.

CIED implantation currently represents one of the few indications for which protective devices are available to reduce post-operative risks^13^. Antibiotic envelopes comprise porous materials and deliver rifampin and minocycline to reach and maintain minimum inhibitory concentration (MIC_90_) in the pocket space for several days after implantation^4^. A large scale randomized clinical study demonstrated that the local and sustained delivery of these classic antibiotics from a resorbable mesh significantly reduces the rate of surgical pocket infections^14^. Whilst successful, these solutions are not equipped to address the surge of antibiotic tolerant or resistant bacterial strains, which are deemed to increase the rate of non-treatable infections^15^. Of particular relevance, in this scenario, are *Staphylococci* (e.g. *S*. *aureus*, *S*. *epidermidis*, etc.) and gram-negative bacteria such as *E. coli*^16^. Natural antimicrobial peptides (AMPs) are present across all types of living organisms and play a crucial role in their innate immune systems. These peptides are known for their broad-spectrum antimicrobial activity, effectively targeting bacteria, viruses, and fungi. Notably, they are less likely to contribute to the development of antimicrobial resistance (AMR) in bacteria and they are emerging as a solution to address this problem^9,10^.

Antimicrobial peptides kill microbes through various mechanisms, primarily by disrupting bacterial membranes. They form pores in the membrane, with models such as the barrel-stave, carpet, and toroidal-pore models explaining the different ways in which AMPs insert and destabilize the membrane. The lipid composition of the bacterial membrane, rich in anionic phospholipids, facilitates the insertion of cationic AMPs, whereas mammalian membranes, containing cholesterol, reduce AMP activity, providing selective toxicity^17^. AMPs’ physicochemical properties, such as hydrophobicity and amphipathicity, influence their membrane interaction, and specific amino acids like proline increase peptide flexibility and activity. In addition to membrane disruption, AMPs can also penetrate bacterial cells to inhibit vital cellular processes, including cell-wall synthesis and protein production. The overall effectiveness of AMPs is shaped by peptide characteristics, environmental factors, and the properties of the target cell membrane^18^.

The SET-M33 peptide, a synthetic non-natural peptide with an amphipathic amino acid sequence closely resembling natural antimicrobial peptides, was developed in preclinical settings^19^. SET-M33 has a tetrabranched structure, obtained using a scaffold of three lysine to support four identical sequence copies attached to their amino groups. This compact dendrimer displays improved stability against proteases, likely due to its larger size, which prevents it from fitting into protease cleavage sites^20^. This increased stability makes it stand out when compared to commonly used antimicrobial peptides (e.g. LL-37) which are susceptible to protease degradation^21^. Furthermore, the multivalent structure of SET-M33 enables stronger interactions with bacterial outer membrane components such as lipopolysaccharide (LPS) and lipoteichoic acid (LTA), leading to faster bacterial killing and beneficial immunomodulatory effects. SET-M33 demonstrated notable MIC_90_ values and effective biofilm eradication against a broad range of bacterial species^22^. SET-M33 exhibits a flexible conformation in aqueous solution but transitions into an amphipathic helical structure upon interacting with SDS micelles, mimicking bacterial membranes, as shown by circular dichroism analysis^23^. This structural change facilitates partial membrane insertion^24^. Investigation of its enantiomers, M33-D and M33-L, using vesicles mimicking *S. aureus* (CL/PG, 4:6) and *E. coli* (PE/PG, 7:3) membranes, revealed a dose-dependent increase in permeability, with M33-D showing slightly higher activity against CL/PG vesicles at higher concentrations^25^. Kinetic studies indicated a biphasic release profile, suggesting a shared membrane-disrupting mechanism independent of lipid composition^25^. Furthermore, scanning electron microscopy of *P. aeruginosa* and *K. pneumoniae* treated with SET-M33 displayed superficial blisters and large membrane disruptions within 60 min, supporting its membrane-targeting activity^26^. Additionally, SET-M33 showed *in vivo* efficacy in treating bacterial sepsis, pneumonia, and skin infections in mice^26^.

With the same principle of anti-fouling modifications, the surface geometry of biomaterials can be employed, together with the chemical-physical composition of the surface, to regulate the interaction with anti-microbial molecules^27^. The biomaterial surface therefore represents a potent parameter to enhance the effect of these drugs both in space and time. At the same time, the porosity of materials employed in protective envelopes controls the diffusion of soluble molecules and represents a key element to support the controlled elution of active chemicals^28^.

Here, we aim at evaluating the efficacy of the SET-M33 antimicrobial peptide in combination with the different implant materials that enter the CIED surgical pocket. The tests presented include the in vitro evaluation of (i) the ability of molecules to permeate porous materials and diffuse in aqueous solution and (ii) to efficiently eradicate bacterial contamination. Altogether, our work identifies viable biomaterials/antimicrobial combinations and indicates the optimal strategy for their deployment in surgical settings.

## Results

### Implantable materials selection

To evaluate the efficacy of the SET-M33 antimicrobial peptide in the prevention of post-operative infections associated with cardiac implantable electronic devices (CIEDs), two groups of materials were included in this study. First, materials that constitute the target implant, including Titanium alloy (**Ti**) for the battery case and silicone rubber (**Si**) for the battery head and lead velour. Polytetrafluoroethylene (**PTFE**) was added to this list as control, representing the standard for anti-adhesive and hydrophobic interfaces^8^.

All the experimental samples were obtained in the form of sterile coupons. The second group included materials used in the production of protective envelopes, such as a non-resorbable membrane made of biosynthesized biocellulose^29^ (**BC**), a resorbable PLGA electrospun membrane^30^ (**Espn**), and a resorbable PGA mesh^31^ (**Mesh**).

The selection included in this study is representative of the CIEDs and protective envelope materials in contact with the surgical pocket tissues. They represent the interface at which bacterial contamination develops in clinical settings. Their specific physical parameters, including porosity, fiber diameter, and/or surface topography and roughness, were similarly defined to reproduce the clinical conditions generated by medical devices currently marketed or in an advanced phase of development for this indication^29,32–34^.

**Fig. 1:**
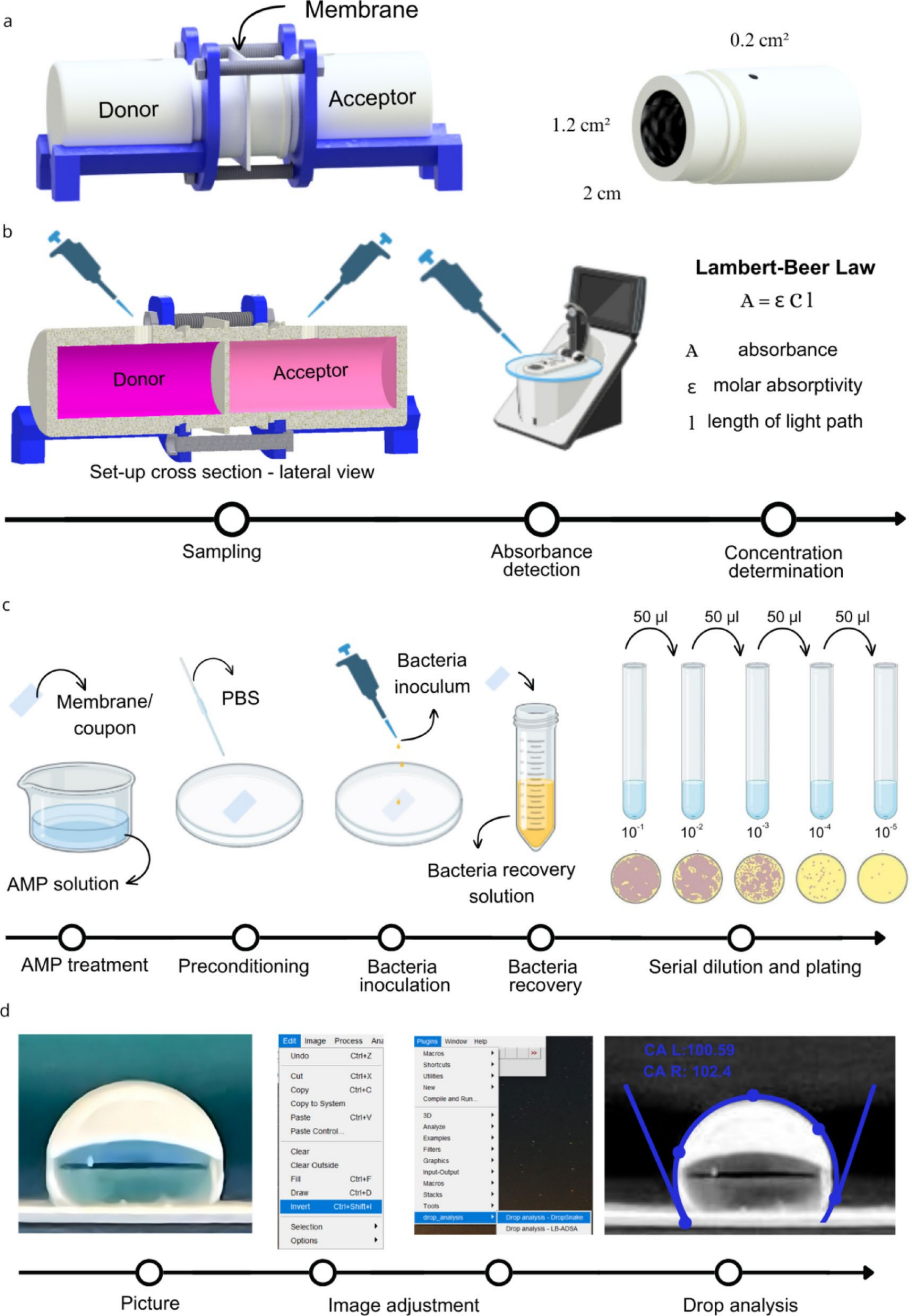
Testing set-ups and protocols are illustrated as follows: (**a**) costum-made side-by-side cell diffusion set up, (**b**) diffusion testing protocol, (**c**) antimicrobial efficiency testing: (1) sample treatment, (2) sample preconditioning, (3) bacteria inoculation, (4) bacteria recovery, (5) enumeration, (**d**) example of image processing for water static contact angle analysis.

The methodologies employed in this study are presented in Fig. 1.

**Fig. 2:**
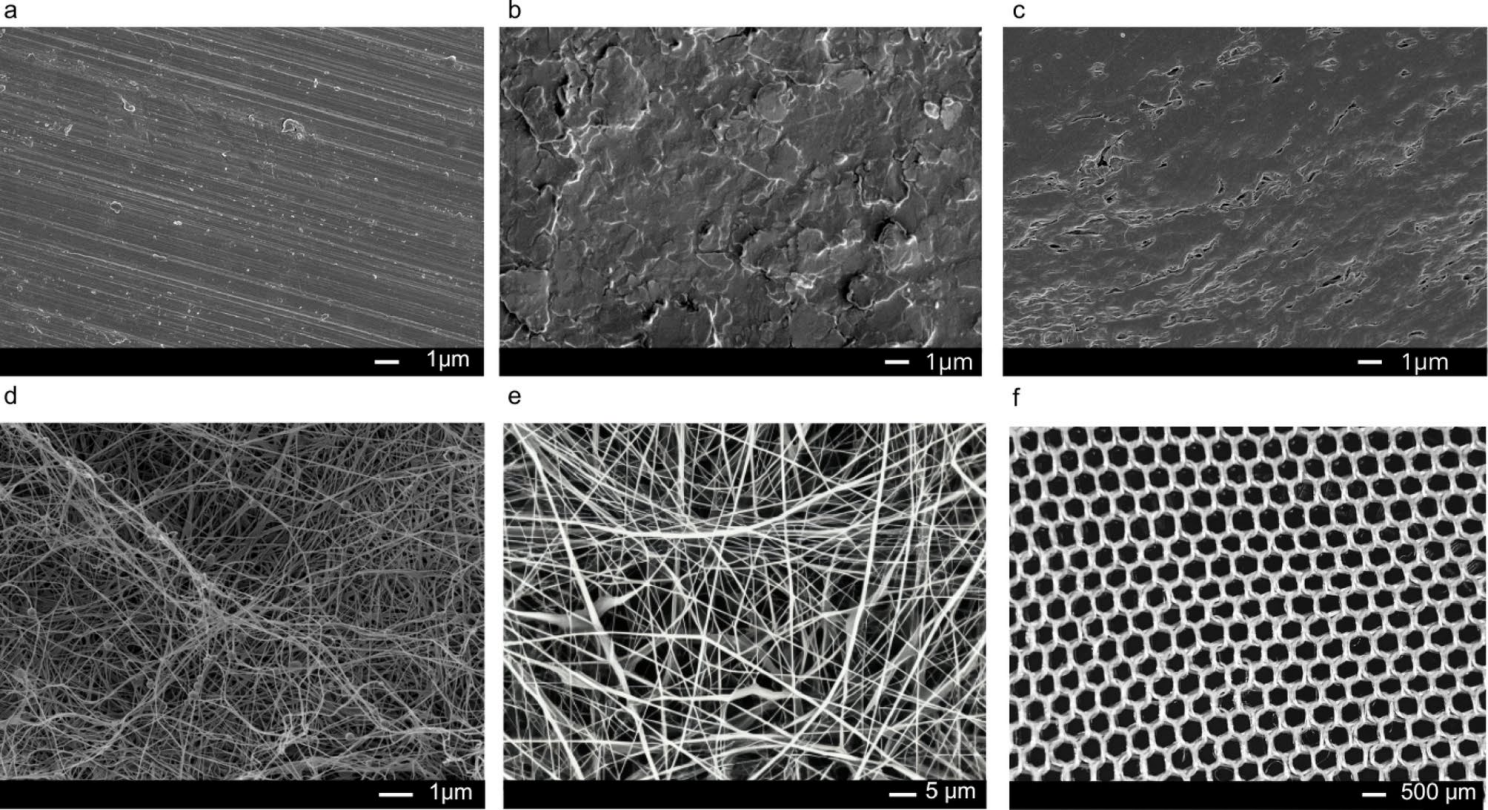
Scanning Electron Microscopy images for: (**a**) Titanium, (**b**) Silicone, (**c**) PTFE, (**d**) Biocellulose, (**e**) Electrospun PLGA, (**f**) Bright field image of warp knitted PGA Mesh 5X.

Since unspecific affinity of antimicrobial peptides to implant materials is governed by physical and chemical properties^31^, including surface roughness, hydrophilicity, porosity, and fiber diameter (for the membrane-based materials) we first analyzed the surface morphology of the implant materials, using scanning electron (SEM) or light microscopy (Fig. 2). The envelope materials, BC and Espn, exhibited a similar interwoven fiber structure (Fig. 2a, b), though they differed significantly in porosity, expressed as a percentage of the total surface area (Table 1). Specifically, the porosity of the Espn (31.03%) was more than double that of the BC (11.98%). The macro-scale surface features of Mesh yielded a 63% porosity (Fig. 2c). The values concerning fiber diameter followed the same trend as those related to porosity. Specifically, BC displayed the smallest fibers (45 ± 0.013 nm) compared to Espn (790 ± 0.17 nm) and Mesh (232 × 10^3^ ± 47 × 10^3^ nm), which represented the material featuring the largest fibers. In contrast, target implant materials did not display regular surface patterns (Fig. 2d-f). Here, the surface geometry was characterized as nano-roughness^35^. PTFE and Ti exhibited comparable R_a_ (see Materials and Methods) values (1.86 ± 0.5 μm and 1.36 ± 0.28 μm, respectively), both significantly higher than Si, whose surface was closer to an ideal smooth form (0.14 ± 0.03 μm, Table 1).

**Table 1:** The table depicts the chemical and physical parameters characterizing the implant and envelopes materials. The first 3 rows show the values related to nano-roughness of Ti, Si and PTFE as µ-Ra, while second three rows list values related to fibers diameter and porosity of the envelope materials. Water static contact angle measurements of all materials are shown in the right column. Values related to all the measurements are presented as mean ± sd among three replicates.

*Implant Materials*	Nano-roughness (µm)		Water Static Contact Angle (°)
PTFE	1.86 ± 0.5		L: 107.35 ± 1.63 R: 109.62 ± 1.75
Ti	1.36 ± 0.28		L: 78.50 ± 0.49 R: 74.80 ± 0.54
Si	0.14 ± 0.03		L: 77.41 ± 0.08 R: 81.92 ± 0.17
Envelope Materials	Fibers Diameter (nm)	Porosity (%)	
BC	45 ± 0.013	11.98	L: 12.66 ± 0.60 R: 12.37 ± 1.93
Espn	790 ± 0.17	31.03	L: 100.05 ± 0.49 R:102.23 ± 0.36
Mesh	(232 ± 47) x 10^3^	63.72	N.A

Next, the water static contact angle (ca., see Materials and Methods) values were measured for each material to evaluate their wettability. The results are presented in Table 1. Among the tested surfaces, PTFE, as expected, proved the most hydrophobic (ca. = 108.48 ± 1.69°) followed by Espn (ca. = 101.14 ± 0.42°), Si (ca. = 79.66 ± 0.12°) and Ti (ca. = 76.65 ± 0.51°). Due to its known hygroscopic properties^36^, BC exhibited very low ca. Finally, the macroscopic architecture of the Mesh, did not allow performing ca. measurements.

### Diffusion properties

Current standard-of-care for the use of protective envelopes in CIED implants requires the homogeneous elution of antibiotic molecules in the surgical pocket to achieve the minimum inhibitory concentration^4^ (MIC_90_). It is therefore critical to analyze the ability of antimicrobial peptides to diffuse across porous envelope materials (BC, Espn, and Mesh).

**Fig. 3:**
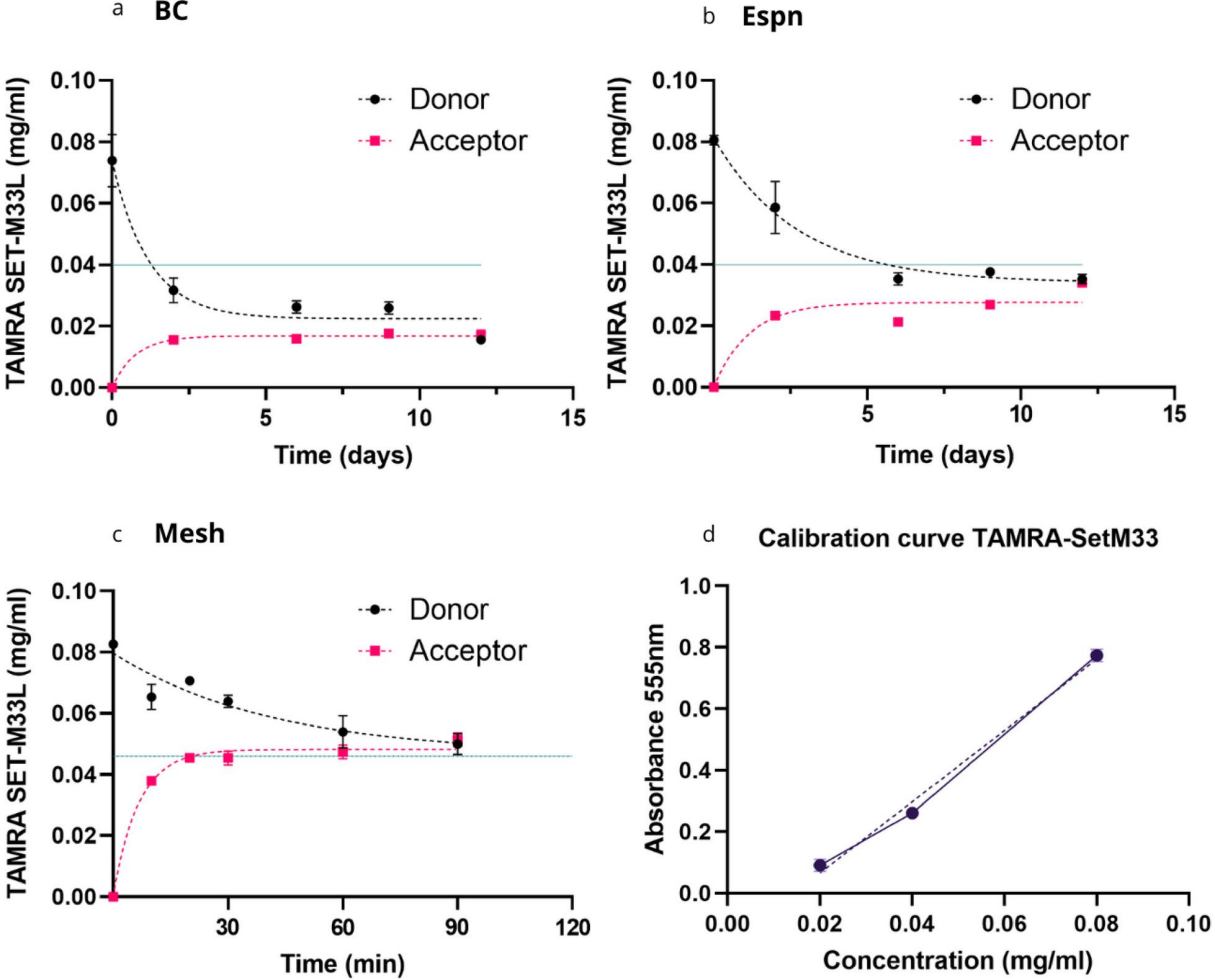
Diffusion trend of TAMRA-SET-M33 across BC (**a**), Espn (**b**), Mesh (**c**). (**d**) The graphs present the mean values of three measurements (*n* = 3) taken from the same sample at each time point, with error bars representing the standard deviation (SD). The dashed lines represent non-linear regression curves indicating the ideal trend of the time points. The continuous line shows half of the initial concentration of the molecule solution added in the Donor compartment. Panel (d) shows the calibration curve of TAMRA-SET-M33. These measurements were consistently replicated across three independent experiments, with one representative experiment depicted in the figure.

To study diffusion of the tetra-branched peptide SET-M33, a TAMRA-rhodamine labelled equivalent was obtained (TAMRA- SET-M33). The diffusion profile for TAMRA SET-M33 across each of the tested porous membranes was measured using a custom designed diffusion system (Figs. 1a and b and 3). From the absorption curves three independent parameters were obtained (Table 2). First, the *diffusion rate*, which indicates the speed at which particles spread or move from an area of higher concentration to an area of lower concentration, i.e. how fast the distribution occurs in the medium^37^. Second, the *effective diffusion coefficient* (D_efy_), which is defined as the rate of transfer of the diffusing substance across unit area considering the complexities and inhomogeneities of the system barriers, porosity, tortuosity, or the presence of multiple phases^38^. Finally, the *drug retention*, which accounts for drug that is retained in the separating membrane and is consequently not released in the acceptor compartment.

**Table 2:** The table presents the diffusion rates and effective diffusion coefficients of TAMRA-SET-M33 across three types of membranes: BC, Espn, and Mesh. The last column shows the percentage of AMP that is retained within the membrane and is no longer released into the liquid phase, indicating a loss of the loaded material. The values provided in the table correspond to one of three independent experiments. Data are expressed as the mean and standard deviation from three measurements conducted on the same sample.

Envelope materials	Diffusion rate (mg/h)	D_eff_ (cm^2^/h)	Drug retain (%)
BC	0.7 × 10^− 3^ ± 0.59 × 10^− 4^	0.0083 ± 0.0005	56.3 ± 1.7
Espn	1.4 × 10^− 3^ ± 0.5 × 10^− 4^	0.003 ± 0.001	14.3 ± 1.7
Mesh	4.126 × 10^− 1^ ± 0.007	1.07 ± 0.11	0

When moving across a BC membrane, TAMRA-SET-M33 concentration in the donor compartment decreased exponentially in the first 2 days, from 0.080 mg/ml to 0.036 mg/ml. An equilibrium between the two compartments was reached after 12 days, yielding values of diffusion rate and D_eff_ of 0.7 × 10^− 3^ ± 0.59 × 10^− 4^ mg/h, and 0.0083 ± 0.0005 cm^2^/h; respectively. Importantly, the displacement of the peptide from the donor compartment did not match a corresponding increase in peptide concentration in the acceptor compartment. Equilibrium between the two compartments was reached at 0.02 mg/ml, indicating that a significant portion of the peptide (56.3% ± 1.7%) was retained within the membrane, as reported in Table 2.

Similarly, the equilibrium between the donor and acceptor compartments was reached after 12 days with the Espn membrane (Fig. 3b), yielding values of diffusion rate and D_eff_ = 1.4 × 10^− 3^ ± 0.5 × 10^− 4^ mg/h and 0.003 ± 0.001 cm^2^/h; respectively. The concentration at equilibrium was reached below the expected value (indicated by the line in Fig. 3b) representing the ideal case at 0.034 mg/ml, indicating that a drug loss of 14.3% ± 1.7 occurred in this case. A different time scale was observed when evaluating Mesh samples, due to their macroscopic porosity (Table 2), with donor and acceptor compartments reaching equilibrium after 90 min (Figure 3c). In addition, Mesh allowed for the almost complete release of the antimicrobial peptide in the acceptor compartment leading to a concentration at equilibrium (0.046 mg/ml) close to the expected value (0.040 mg/ml). Altogether, these data indicate that, for the tested materials, the diffusion rate positively correlates with the membrane porosity whereas the overall drug retain has the opposite trend. A large proportion of the drug was retained by BC membranes.

### Antimicrobial efficacy

Next, the antimicrobial performance of the SET-M33 peptide in combination with the selected implant materials was evaluated through in vitro bacterial challenge tests against *S. aureus* and *E. coli*. These bacteria were selected as representing the most frequent gram-positive and gram-negative pathogens in CIED pocket infections^16^. Two versions of SET-M33 were used. SET-M33L, prepared will all-L configuration aminoacids and SET-M33D obtained using D-aminoacids^39^. Primarily, the treatment with antimicrobial peptide SET-M33 caused an increased surface hydrophilicity in all the materials employed in the study (Supplementary Table S1).

To test whether the combination of the selected materials with the SET-M33 introduces additional safety concerns, a cytotoxicity test was performed using the THP-1 human monocytic cell line (Supplementary Figure S1I). These cells were selected as representing an established model for in vitro cytotoxicity tests^40^. All tested materials demonstrated optimal compatibility yielding cell viability above 90%.

The combination with SET-M33 did not introduce additional cytotoxicity and the viability remained comparable in all tested conditions (Supplementary Figure S1 II). These results indicate that the selected materials alone and in combination with SET-M33 demonstrate good biocompatibility.

**Fig. 4:**
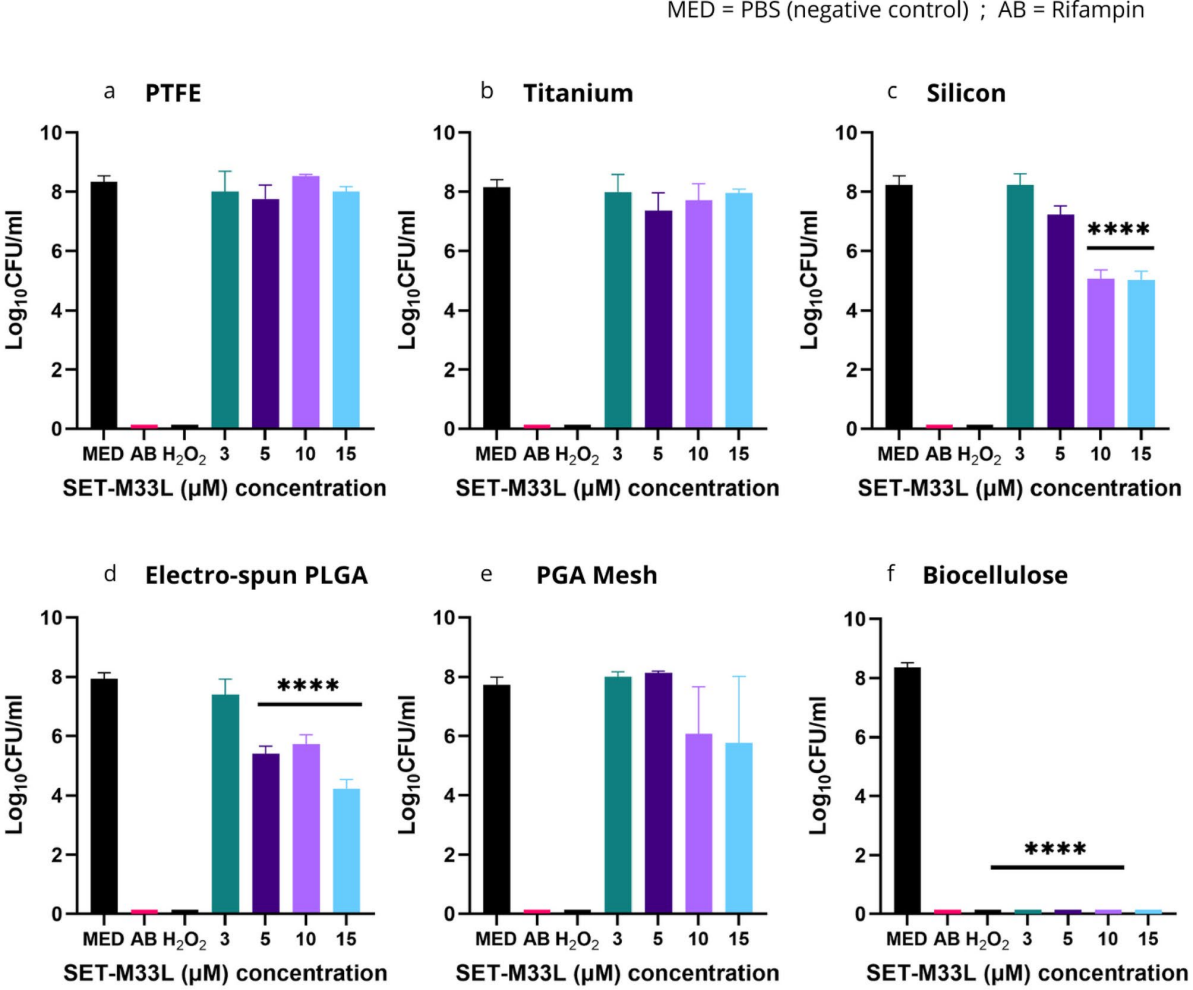
Growth of *E. coli* on PTFE (**a**), Ti (**b**), Si (**c**), Espn (**d**), Mesh (**e**) and BC (**f**) treated with increasing concentrations of SET-M33L, compared to untreated, Rifampin and H_2_O_2_ treated controls. Bars indicate mean and standard deviation (SD) among three replicates for each sample. Each graph represents one of three independent experiments that produced consistent results. **** Represents significant difference (*p* < 0.0001) between the treated sample and the untreated control.

Figure 4 displays the growth of *E. coli* on untreated and SET-M33L-treated samples after 24 h incubation. The growth of *E. coli* on non-treated samples was comparable on all tested surfaces indicating that no substrate-based effect could be measured (average growth on non-treated samples was 8.33 Log_10_ CFU/ml for PTFE, 8.15 Log_10_ CFU/ml for Ti, 8.23 Log_10_ CFU/ml for Si, 7.93 Log_10_ CFU/ml for Espn, 7.73 Log_10_ CFU/ml for Mesh, and 8.36 Log_10_ CFU/ml for BC; Fig. 4a-f). For the target implant materials, interaction of SET-M33L at all tested concentrations (3µM, 5µM, 10µM, 15µM) with PTFE and Ti substrates did not show any significant effect in reducing *E. coli* growth (Fig. 4a-b). On the other hand, bacterial growth on Si was reduced at 10 µM (5.06 Log_10_ CFU/ml) and 15 µM (5.03 Log_10_ CFU/ml), corresponding to 3x and 5x previously established MIC (Fig. 4c). The antimicrobial activity of SET-M33 on the implant materials was compared to the positive outcome of Rifampin treatment that inhibited the bacteria growth in all the surfaces and was indicated as the positive control in the experiment.

For the envelope materials (Fig. 4d-f), a different trend was observed. With the increasing concentration of SET-M33, the growth of *E. coli* on Espn decreased linearly, leading to a significant reduction at 15 µM peptide (4.23 Log_10_ CFU/ml vs. 7.93 Log_10_ CFU/ml. Figure 4d). On the other hand, the treatment with SET-M33L did not have any measurable effect on bacterial growth on Mesh samples at any of the tested concentrations (Fig. 4e). Remarkably, a complete growth inhibition was obtained on BC membranes at all tested concentrations (Fig. 4f).

**Fig. 5:**
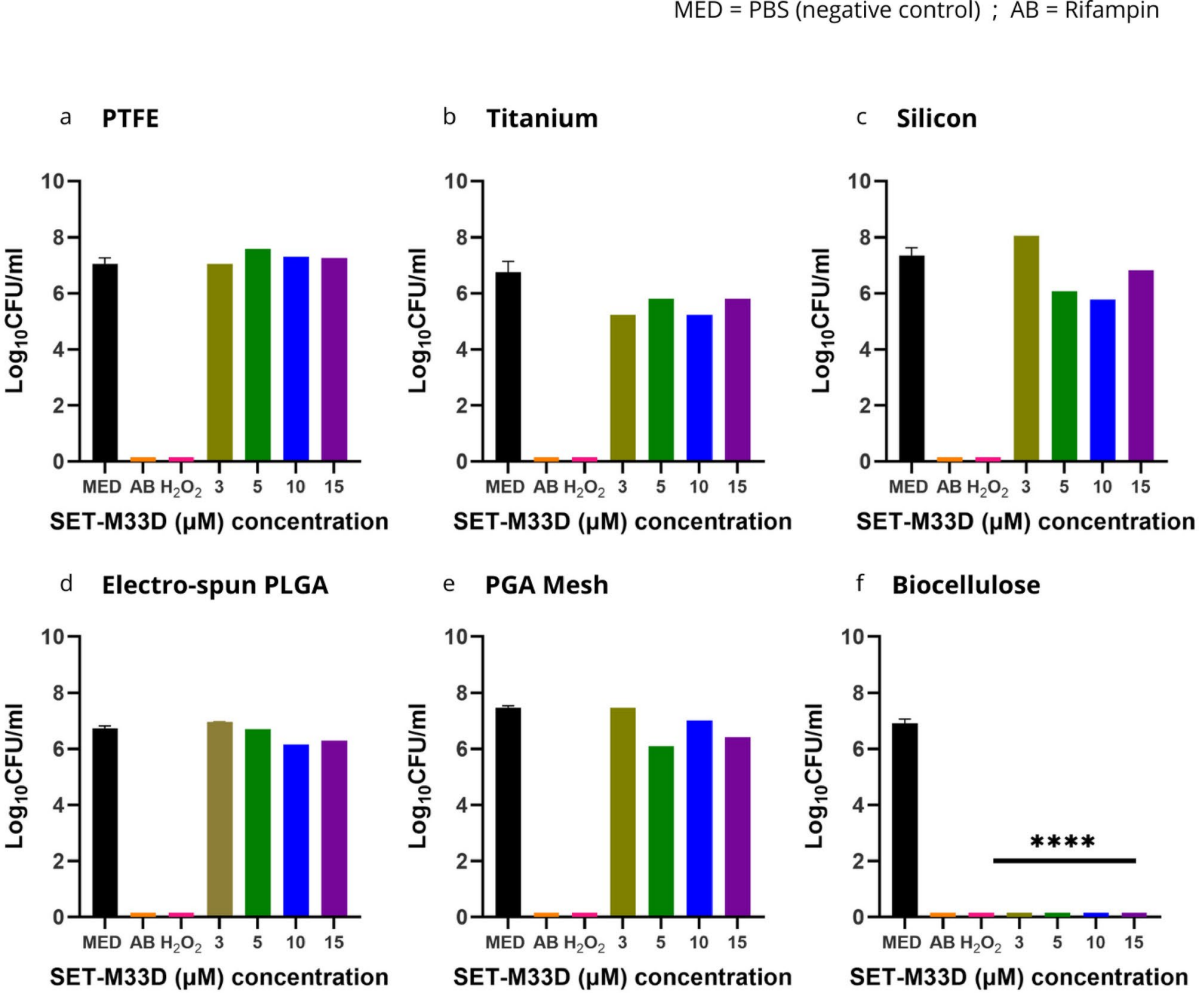
Growth of *S. aureus* on PTFE (**a**), Ti (**b**), Si (**c**), Espn (d), Mesh (e) and BC (f) treated with increasing concentrations of SET-M33L, compared to untreated, Rifampin and H_2_O_2_ treated controls. Bars indicate mean and standard deviation (SD) among three replicates for each sample. Each graph represents one of three independent experiments that produced consistent results. **** Represents significant difference (*p* < 0.0001) between the treated sample and the untreated control.

Comparable results were obtained when SET-M33D was challenged in combination with all the tested materials against *S. aureus (*Fig. 5a-f). No antimicrobial effect was detected on PTFE, Ti or Si (Log_10_ CFU/ml recovered were 7.05 Log_10_ CFU/ml for PTFE, 6.75 Log_10_ CFU/ml for Ti, and 7.34 Log_10_ CFU/ml for Si; respectively. Figure 5a-c). Similarly, Espn and Mesh substrates treated with SET-M33 had no significant effect on bacterial growth at any of the tested concentrations (Fig. 5d-e). On the contrary, when combined with BC, SET-M33 could fully eradicate bacteria at all tested concentration (Fig. 5f). Colony growth on agar plates for all the tested conditions is shown in the Supplementary Information (Supplementary Figure S3).

In light of the limited diffusion rate and high drug retention demonstrated by the SET-M33 across BC membranes (Fig. 3a), the efficacy of this combination was further evaluated. Specifically, an alternative configuration for the bacterial challenge was tested. While in the experiments reported in Figs. 4 and 5, the antimicrobial peptide and the challenging bacteria were applied to the same side of the BC substrates (*cis* configuration), in these furthers tests, the antimicrobial peptide and the challenging bacteria were applied to opposite sides (*trans* configuration) of the BC membrane. We previously demonstrated that bacteria cannot cross BC membranes^29^, however the efficacy of SET-M33 in these settings remains to be addressed and is bound to its ability to cross the BC membrane.

**Fig. 6:**
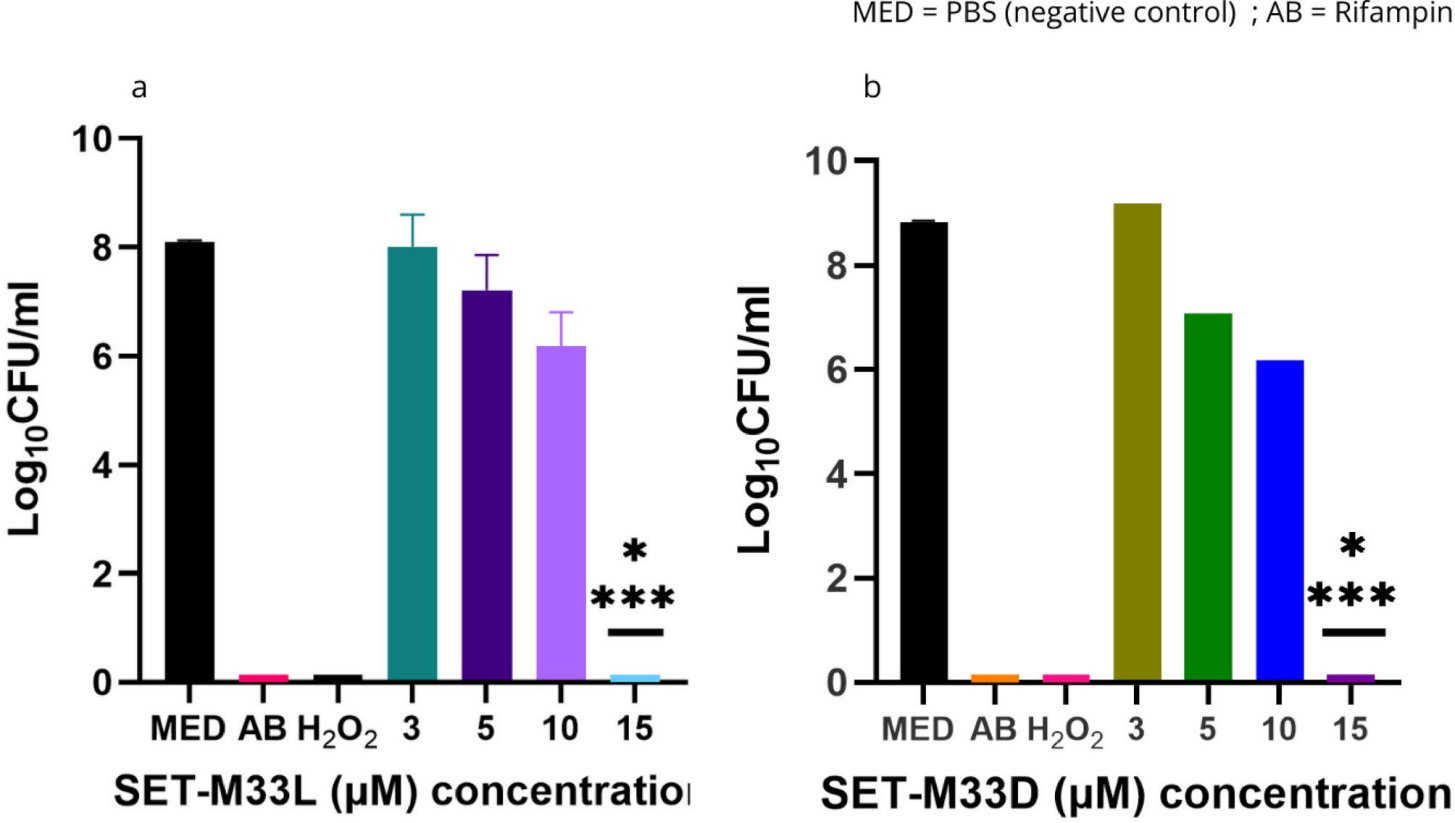
Growth of *E. coli* (a) and *S. aureus* (b) on BC when bacteria inoculation is performed on the opposite side of the sample with respect to the antimicrobial treatment. Antimicrobial peptide treatment is compared to untreated controls, Rifampin and H_2_O_2_ treated samples. Bars indicate the mean and standard deviation (SD) among three replicates for each sample. The results presented refer to one of two independent experiments performed that showed consistent results.

In the trans configuration, a marked reduction of antimicrobial efficacy against *E. coli*, was observed (Fig. 6a). At the lowest peptide concentrations, specifically 3 µM, 5 µM, and 10 µM, the AMP treatment exhibited limited effectiveness, with average colony counts of 7.19 Log10 CFU/ml, 6.8 Log10 CFU/ml, and 4.12 Log10 CFU/ml; respectively (vs. 8.09 Log10 CFU/ml in untreated). Complete inhibition of bacterial growth was only achieved at the highest tested concentration (15 µM). A similar trend was observed against *S. aureus*, as shown in Fig. 6b. These results demonstrate that the limited diffusion and high retention of the SET-M33 peptide across BC membranes hinders its efficacy against bacteria growing at the non-treated interface.

As control, the materials were treated with hydrogen peroxide (H₂O₂). H₂O₂ is routinely used for washing the CIED surgical pocket and the implant upon CIEDs implantation^41^. All tested materials were treated with a 3% H₂O₂ solution. The treatment was effective in erasing bacterial growth on all tested materials, when challenged with* E. coli* or *S. aureus* (Figure 4, 5). Interestingly, full efficacy was also observed when BC substrates were challenged in the trans configuration (Figure 6). To extend these conclusions to experimental conditions allowing the formation of biofilm, anti-biofilm assays were conducted using a genetically modified, bioluminescent *E. coli* strain. This strain enables the visualization and quantification of high- and low-density biofilm regions on biomaterial surfaces^42^.

The results (Supplementary Figure S3) confirm that the SET-M33L treatment exerted no detectable antibiofilm effect on the tested substrates, with the exception of BC. Here, a significant inhibition of both bacterial adhesion and biofilm formation was observed at all tested concentrations when the SET-M33 was combined in the cis configuration. On the other hand, a significant inhibitory effect on biofilm formation was only observed at the highest tested concentration (15 µM) when SET-M33 was combined to BC substrates in the trans configuration. These results confirm the findings obtained on planktonic bacteria (Fig. 6).

## Discussion

The study investigates the interaction of the synthetic antimicrobial peptide SET-M33 with various implant materials used in cardiac implantable electronic devices (CIEDs) and protective envelopes. It highlights that porous biomaterials like BC and electrospun membranes facilitate peptide diffusion, enabling effective antimicrobial activity against pathogens such as *E. coli* and *S. aureus*. However, non-porous materials like titanium and silicone limit peptide efficacy. Notably, while BC retains a significant portion of the peptide, hindering its availability at distant sites, its ability to eradicate bacterial growth on contact demonstrates promise for targeted infection prevention.

The findings underline the critical role of material properties—such as porosity and surface morphology—in modulating peptide performance, emphasizing the need for tailored approaches in implant care. While current antibiotic-eluting envelopes offer infection control, the study advocates for optimized antimicrobial peptide delivery systems. These innovations could address growing antibiotic resistance by providing prolonged, localized infection prevention, potentially transforming postoperative care for CIED recipients. The current standard of care for the prevention of pocket infections in high risk CIED and neurostimulator patients recommends the use of antibiotic envelopes. These protective devices comprise a polymeric mesh which enforces the release of two antibiotic molecules in the surgical pocket for several days after implant^31^. To attain optimal protection, the antibiotics shall diffuse rapidly and homogeneously reaching a minimum inhibitory concentration in the pocket space, including the surrounding soft tissues and the implant interface. This aspect is key, as simple washes of the surgical pocket with antibiotic or antimicrobial solutions, performed during the surgical procedure, do not provide the same preventive benefit^43,44^. Looking at the future of infection prevention in implant recipients, the local and continued release of antimicrobial molecules shall therefore provide equivalent protection without eliciting antibiotic resistance. In this frame, the use of antimicrobial peptides opens the possibility to ensure long-term coverage extended to several weeks post implant, addressing the rise of late surgical pocket infections^14^. Sustained and homogeneous availability of an effective peptide concentration over space and time will necessarily depend on the complex interaction between the implant materials and the selected molecules. Two alternative solutions can be envisaged. First, the inclusion of antimicrobial peptides in protective envelopes similar to those currently available^3132^,that are assembled around the target implant before deployment. Second, the direct coating of the implant surface, including the battery case and leads. Both options carry intrinsic technical challenges.

Materials comprising the interface of electronic implants, including Titanium and Silicone, do not support the antimicrobial activity of the SET-M33 against *S. aureus* and *E. coli* in our experimental settings. These substrates are selected with a functional intent. By design, they are non-porous and relatively smooth with the intention to limit fouling. It is therefore to be expected that the simple coating of titanium and silicone is not sufficient to support the activity of the SET-M33 peptide. Variations in surface roughness, chemistry and/or wettability of these materials, which may facilitate coating, are not simply implemented. Any such modification would bring into question the overall device functionality. Furthermore, the stochastic nature of SET-M33 peptide surface adhesion influences its structural conformation and affects the material surface nature. As antimicrobial activity is intrinsically dependent on peptide structure, uncontrolled interactions with material surfaces may attenuate its efficacy. Along this line, the only viable development shall envision the chemical engineering of the peptide itself, to include functional groups allowing controlled interaction with the target surface, without compromising the antimicrobial effect.

Fibrous protective envelopes display high porosity, which depends on the fiber size and architecture, rendering them conducive to SET-M33 adsorption and diffusion. The combination of these two processes supports the antimicrobial activity of the peptide in bacterial challenge tests. Altogether, more flexibility is possible in the selection of excipients and architectures for the protective envelopes. The structure of these devices is porous, however with variations which are dependent on the fabrication method. Porosity and chemical composition define the ability of soluble molecules to cross the intervening layer formed by the envelopes between the target implant and the surgical pocket tissues. For optimal distribution, large molecules such as peptides should diffuse rapidly, typically in the first hours after implants. This is granted by structures with macroscopic pores, such as meshes. Envisioning a simple loading of an antimicrobial solutions into the matrix of an envelope material, shall leverage the loading capacity of the matrix itself. This is very high in hydrogels such as biosynthesized cellulose and can lead to more effective antimicrobial protection.

On the other hand, drug retention must be considered as a factor influencing the antimicrobial efficacy of biomaterial-peptide combination. Drug retention in the selected porous materials has different implications towards the clinical use in protective envelopes. BC is non-resorbable^29^ and protective BC envelopes are conceived to enforce a durable intervening layer^6^. The average pore size of the BC layer impedes the penetration of bacteria, which are confined to its surface^29^. Antimicrobial peptides which are stably retained in the BC matrix may therefore not contribute to the antibacterial effect (Fig. 6). In this scenario, high drug retention hinders the potential efficacy of AMPs in combination with BC. On the other hand, smaller antimicrobial molecules, which have a diffusion coefficient similar to water, are able to exert full activity across BC membranes. Diffusion and retention are therefore important parameters determining the effective availability of antimicrobials in combination with BC. Resorbable materials (Mesh and Electrospun) still display a small fraction of peptide retention (Table 2). Upon implantation, the fibers (e.g. PLGA, PGA, PEG, and similar) undergo degradation by hydrolysis^45,46^. This process induces additional pore formation, followed by fragmentation^47,48^. The drug initially retained in the matrix shall therefore be released over time, as demonstrated by marketed products featuring in vivo elution over several days^4^. This mechanism opens therefore to the sustained release of AMPs loaded in resorbable porous materials.

In summary, it appears that the current state of development for antimicrobial peptides, which are optimized for the protection of surfaces from biofilm formation^25^, will not be immediately translated into off-the-shelf solutions for the postoperative protection of surgical pockets hosting permanent implants. Dedicated peptide configurations will be required, with modifications ensuring optimal efficacy and prolonged and homogeneous coverage in the complex environment of the surgical pocket. To effectively address the critical challenge of antibiotic-induced microbial resistance in surgical pocket care, this paper’s findings lay the groundwork for the development of next-generation antimicrobial drugs. Antimicrobial peptides may offer a promising alternative to antibiotics for surgical environments, if tailored to match—and ultimately surpass—the efficacy of current antibiotic envelopes, offering a more sustainable and resistant-free solution for infection prevention in implant care.

## Methods

For the presented experiment the tested implant materials were divided into two groups: Permeable materials: Materials with porosity allowing for percolation of soluble molecules. Biocellulose membrane (BC), Electrospun PGA/PLGA membrane (Espn) and PGA Mesh in the form of rectangular samples of 4 × 3 cm. Non-permeable materials: Titanium (Ti), Silicon (Si) and Polytetrafluoroethylene (PTFE) in the form of implantable standardized sterile coupons of 12.7 mm diameter x 3.8 mm height (RD128-Ti6AL, RD128-Si, RD128-PTFE, BioSurface Technologies).

### Scanning electron microscopy

Samples were sputter coated with 4 nm Pt/Pd and images were acquired with TFS Magellan 400 (Thermo-Fisher Scientific) with field emission 200 kV via secondary electron detection. To image BC membranes samples were previously dried via critical point drying.

### Light microscopy analysis

Bright field image was taken with Leica DMi8 microscope with integrated camera, using 5X objective.

### Image analysis

SEM images were analyzed to extract physical parameters relative to envelope materials. The porosity and fiber diameter were extrapolated via imaging analysis using ImageJ (National Institute of Health, US), as follows.

*Porosity measurement.* To assess porosity, the images were first converted to 8-bit format. The “Threshold” function was the applied. Specifically, the pixel intensity distribution varied until all pores were captured. Subsequently, using the “Analyze” menu, the “Measure” function was selected, and porosity was calculated as a percentage of the total image area.

*Fiber Diameter Measurement.* Fiber diameter was quantified importing SEM images. The scale was set via the “Set Scale” function. For each fiber (*n* > 15), a line was drawn across its diameter, ensuring the endpoints were aligned with the fiber edges. The “Measure” function was then employed to determine the fiber diameter based on the traced line.

### Surface roughness

Surface roughness measurements were conducted at RT using a calibrated MAHR PS-1 Surface Roughness Measuring Instrument (SFS, Switzerland). The measurements were performed in accordance with ISO 4288 and ASME B46.1 standards, with a cutoff length of 1.75 mm. In regions where sanding grain was detectable, measurements were taken perpendicular to the grain direction to ensure accuracy. Surface roughness is quantified by the deviations in the direction of the normal vector of a real surface from its ideal form and measured by the parameter Ra: the average of how far each point on the surface deviates in height from the mean height.

### Water static contact angle

Hydrophilicity of material surfaces was measured via water static contact angle analysis. Specifically, a controlled volume (20 µl) of DI water was carefully placed on the surface of each material. Firstly, the samples were washed by immersion and sonication for 5 min in DI water, followed by isopropanol and again DI water and dried under nitrogen flow. Each sample was placed on a flat surface, against a dark background to enhance the visibility of the object’s contours in the image. Pictures were taken with ƒ/1.6 aperture camera equipped with macro lens and saved as RGB color. Each image was analyzed with the software ImageJ. The RGB image was split in the blue, red and green channel and the red color channel was selected for the analysis. The image was further processed using the function “invert’ to provide a better visualization of the drop against the background. The water static contact angle (c.a.) was measured using the drop analysis plug in of ImageJ defining the drop contours and allowing the software for the fitting and calculation of the internal angles that define the hydrophilicity/hydrophobicity of the surface. Figure 1d provides an example of processing. A c.a. below 90° indicates hydrophilicity, while values above this threshold denote hydrophobicity.

### Peptide synthesis

The peptides SET-M33D (kkirvrlsa)_4_K2KβA-OH and SET-M33L (KKIRVRLSA)_4_K2KβA-OH were solid-phase synthesized by standard Fmoc chemistry using D-amino acids with a Syro multiple-peptide synthesizer (MultiSynTech, Witten, Germany), as previously described^22^. TentaGel-PHB 4 branch βAla Wang-type resin (Rapp Polymere, Germany) was used, which carries the branching core in L-form, Fmoc4-Lys2-Lys-β-Ala.TAMRA-SET-M33 was synthesized using FmocLys(TAMRA)OH as first aminoacid, FmocPEG4-OH as second coupling step and FmocLys(Fmoc)OH as third and fourth coupling steps. Sidechain-protecting groups were 2,2,4,6,7- pentamethyldihydrobenzofuran-5-sulfonyl for R, t-butoxycarbonyl for K, and t-butyl for S. The final product was cleaved from solid support, deprotected by treatment with TFA containing triisopropylsilane and water (95/2.5/2.5), and precipitated with diethyl ether. Crude peptide was purified by reversed-phase chromatography on a Phenomenex Jupiter C18 column (300 Å, 10 mm, 250, 610 mm), using 0.1% TFA/water as eluent A and methanol as eluent B, in a linear gradient from 80% A to 20% A in 30 min. Final peptide purity and identity were confirmed by reversed-phase chromatography on a Phenomenex Jupiter C18 analytical column (300 Å, 5 mm), and by mass spectrometry with a Bruker Daltonics Ultraflex MALDI TOF/TOF).

### Diffusion testing

For the diffusion tests a costum-made side-by-side cell diffusion set-up (Fig. 1a) was built assembling two 50 ml LDPE bottles provided with a 1.2 cm^2^ aperture on the screw cap face and a 0.2 cm^2^ aperture on the side. The bottles were aligned horizontally with the two openings facing each other, creating two separate chambers. The system was sealed using a 3D printed PLA support together with a screwing system.

For each test, a 3 × 3 cm membrane sample was cut and placed between the two apertures. An O-ring was used to ensure a complete seal, preventing leakage. One of the two chambers was designated as Donor compartment and filled with 12 ml TAMRA-SET-M33 PBS solution (0.08 mg/ml), while the second chamber, identified as the Recipient was filled with a blank solution of PBS. Magnetic bars were included in both chambers and the system was incubated at 37 °C on a stirring plate. To measure diffusion, a calibration curve for TAMRA- SET-M33 dissolved in PBS was initially determined using a Nanodrop One Microvolume UV-Vis spectrophotometer (Thermo-Fisher Scientific) to measure absorbance at ʎ = 555 nm. The Lambert-Beer law was then used to convert absorbance of each sample in a concentration value (mg/ml). The corresponding protocol is shown in Fig. 3b. Based on the calibration curve (Fig. 3d), the time to equilibrium was measured and the diffusion rate and effective diffusion coefficient (D_eff_) were obtained for each diffusion experiment. Specifically, to evaluate the diffusion dynamics, the chambers were sampled every 2 days. Individual samples of 100 µl were collected and absorbance at ʎ=555 nm was immediately measured.

### Bacterial culture

Reference strains of *E. coli* ATCC25922 and *S. aureus* ATCC29213 were grown o/n on nutrient Luria Bertani (LB) agar. Due to 20 min doubling time of *E. coli* in favorable conditions^48^ and 20 min doubling time for *S. aureus*^49^ o/n incubation is sufficient to assess bacterial growth. Isolated colonies were harvested and resuspended in LB broth adjusting the volume to reach OD600 of 0.1, corresponding to 1.5 × 10^8^ CFU/ml. Stock solution was diluted to produce a final concentration of 1.5 × 10^5^ CFU/ml and a working concentration of 1.5 × 10^4^ CFU/ml.

### Antimicrobial efficacy of AMPs in combination with implant materials

The antimicrobial efficacy of SET-M33 in combination with implantable materials was assessed adapting the AATCC TM100:2019 protocol (Fig. 1c). Two isomeric forms of the SET-M33 peptide, SET-M33L and SET-M33D, are available. Whilst their molecular weight and steric hindrance are identical (MW: 5857 g/mol), their specific efficacy is strain-dependent^25^. Both isomers exhibited comparable efficacy against *E. coli* with a minimum inhibitory concentration (MIC) of 3 µM. However, the D isomer demonstrated superior efficacy against *S. aureus*, with lower MIC to 1.5 µM^25^. Therefore, the L-isomer was used in the tests against *E. coli*, whereas the D-isomer was adopted against *S. aureus*.

For efficacy experiments, implant materials and membranes materials samples were first immersed for 1 h at RT (for BC overnight) in a SET-M33 solution in PBS at concentration of 3 µM, 5 µM, 10 µM, 15 µM. They were then collected from the solution, rinsed in PBS and placed in a Petri dish where 2 ml of PBS were added around the sample to ensure a moist environment. Bacterial suspensions (*E. coli* or *S. aureus*) were added on top of the samples, dropwise in a five dice arrangement. The inoculation volume (i.e. 100 µl for permeable materials and 50 µl for non-permeable materials with 1,5 × 10^5^ CFU/ml) was chosen to ensure a uniform distribution of the bacteria given the difference in surface area. A negative control (equivalent sample immersed in PBS) and a positive control (equivalent sample immersed for 1 h in a 60 µg/ml rifampin solution) were tested together with AMP treated samples. Samples were incubated at 37 °C in a humidified environment o/n. Controls and test samples, along with the residual preconditioning fluid, were then harvested, moved into a 15 mL tube containing 10 mL PBS and vortexed for 5 min. The bacteria in the solution were enumerated via serial dilution and plating on nutrient LB agar.

### Bacterial colony data quantification and statistical analysis

For quantification of bacteria, the average Log_10_ CFU/ml were reported as mean ± standard deviation (SD) from three independent experiments. In Figs. 4 and 5 the histogram bin represents the mean across the replicates of the experiment, while the error bars refer to standard deviation across the same replicates. Statistical analyses were performed using Prism 9.0 (GraphPad Software Inc., San Diego, CA, USA) and one-way ANOVA test was used to assess statistical differences between the Log_10_ CFU/ml recovery data from negative control and test items. Difference was considered statistically significant at *p* < 0.0001. The test outcome was considered significant if the reduction factor between non-treated and treated samples was ≥ Log_4_.

## Electronic supplementary material

Below is the link to the electronic supplementary material.

Supplementary Material 1

## Data Availability

All data generated and analyzed during the current study are available from the corresponding author on a reasonable request.
