# Supplementary material for: The identity of implant materials governs the antimicrobial efficacy of SET-M33
Source: Sci Rep. 2025 May 10;15:16353. doi: 10.1038/s41598-025-99808-w (PMC12065777; doi:10.1038/s41598-025-99808-w)
Supplement: Supplementary file 1 — Supplementary Material 1 [file 41598_2025_99808_MOESM1_ESM.pdf]

Supplementary Information

Supplementary methods

Cells viability assay on SET-M33 treated biomaterials

The biocompatibility of SET-M33L-treated biomaterials was evaluated using the THP-1 human monocytic cell line. BC, Mesh, ESPN (1×1 cm samples), and Ti and Si disks were treated in a 24-well plate with either PBS or 15 µM SET-M33L for 4 hours. Following treatment, samples were washed with 1 mL PBS, and 8×10^4 THP-1 cells were seeded directly onto the biomaterial surfaces. The plate was incubated at 37°C with 5% CO_2 for 24 hours to allow cell attachment and interaction with the treated materials.

After incubation, cells in each well were thoroughly resuspended by pipetting to ensure detachment from the biomaterial surfaces. Samples were then removed, and the collected cells were centrifuged at 1400 RPM for 5 min at 4°C. The cell pellet was stained with the Live/Dead Fixable Violet Dead Cell Stain Kit (Invitrogen) in the dark for 10 min. Cells were washed by centrifugation, the supernatant was discarded, and the pellet was resuspended in 200 µL FACS buffer. After an additional wash step, cells were fixed by resuspension in 100 µL fixation buffer and incubated for 15 min in the dark at room temperature. A final centrifugation step was performed, the supernatant was discarded, and the pellet was resuspended in 250 µL FACS buffer. Cell viability was assessed using a BD FACS Canto II flow cytometry system, and data were analyzed with FlowJo software.

Preparation of bioluminescent *E. coli* strain

Cloning and transforming the plasmid: The vectors pcrRNA.con (Addgene #61285), ATG-3925 (Addgene #167839), and miniSOG-C1 (#54821) were used to generate the final plasmid expressing CBG2 and miniSOG under the *J23119* constitutive promoter. The primers used are listed in Supplementary Table S1. All PCR reactions were performed using the Phusion™ High-Fidelity DNA Polymerase (Invitrogen), following the manufacturer's protocol.

Three intermediate plasmids were generated through Gibson Assembly using the GeneArt™ Gibson Assembly HiFi Master Mix (Invitrogen): one containing the *CBG2* gene (*JCrT1*), a second containing the *miniSOG* gene (*JmrT1*), and a third in which the ampicillin resistance gene in *JmrT1* was replaced with a kanamycin resistance gene (*JmrT1 Kan*). A Shine-Dalgarno sequence ("TAAGGAGGT") was inserted between the *J23119* promoter and the *CBG2/miniSOG* sequences. Additionally, an HA tag and a stop codon were added to the *miniSOG* sequence.

The final construct, *JCmrT1 Kan*, containing *CBG2*, *miniSOG*, and a kanamycin resistance gene, was generated by incorporating *BamHI* and *XbaI* restriction sites via PCR, followed by restriction digestion and ligation using the T4 DNA Ligase (Promega). The resulting plasmids were transformed into DH10B Electrocompetent Cells (Invitrogen) using 0.1 cm Gene Pulser/MicroPulser Electroporation Cuvettes (BioRad) and the Gene Pulser BioRad Electroporator (BioRad). Electroporation was performed at 1.7 kV, 200 Ω resistance, and 25 µF capacitance. Bacteria were recovered for 1 hour at 37°C, then plated on LB/agar supplemented with 100 µg/mL ampicillin or 50 µg/mL kanamycin. Different clones were selected, and whole plasmid sequencing was performed by Plasmidsaurus (OR, USA) using Oxford Nanopore Technology with custom analysis and annotation.

Preparation of Electrocompetent E. coli Seattle 1946: Electrocompetent *E. coli* Seattle 1946 (ATCC25922 / DSM 1103) cells were prepared following standard bacterial culture and cryopreservation protocols. A single colony from an LB/agar plate was inoculated into 5 mL of LB medium and incubated overnight (~16 h) at 37°C with shaking. The following day, 5 mL of the overnight culture was transferred into 500 mL of fresh LB medium and grown to an OD600 of 0.4–0.6 to ensure cells remained in the mid-log phase. The culture was then placed on ice water for 15 minutes to cool.

Cells were harvested by centrifugation at 4,000 rpm for 10 minutes at 4°C. The pellet was resuspended in 100 mL of ice-cold 10% glycerol (or dH_2O as an alternative) and incubated on ice for 5 minutes. A second centrifugation was performed at 4,000 rpm for 5 minutes at 4°C, followed by resuspension in 20 mL of 10% glycerol. This washing step was repeated with another centrifugation at 4,000 rpm for 5 minutes at 4°C, and the final pellet was resuspended in 2 mL of 10% glycerol.

Aliquots of 50 µL were prepared in sterile microcentrifuge tubes, flash-frozen on dry ice, and stored at -80°C for future use.

Anti-biofilm experiment with bioluminescent *E. coli*

E. Coli biofilm formation follows a stereotyped dynamic, with initial surface adhesion occurring within minutes to hours, microcolony formation by 6–12 h, and biofilm maturation progressing over 12–48 h[51]. Specifically, the culture medium was refreshed 6 h after bacterial challenge to support biofilm formation. The biofilm was then allowed to form on the material samples overnight. To eliminate residual planktonic bacteria, samples were carefully washed with PBS before bioluminescence analysis [52]. D-Luciferin was added to the samples to trigger the bioluminescence reaction. Imaging was performed using the IVIS Spectrum Imaging system, specifically visualizing the biofilm and enabling precise identification of high-density biofilm regions on the surface[51]. Bioluminescence intensity was quantified based on photon emission. For each sample, the emission was measured within a defined Region of Interest (ROI), and the number of photons emitted per second was calculated using

the measurement tool of ImageJ. The bioluminescence intensity of SET-M33-treated samples was compared to that of the untreated control samples.

Supplementary figures

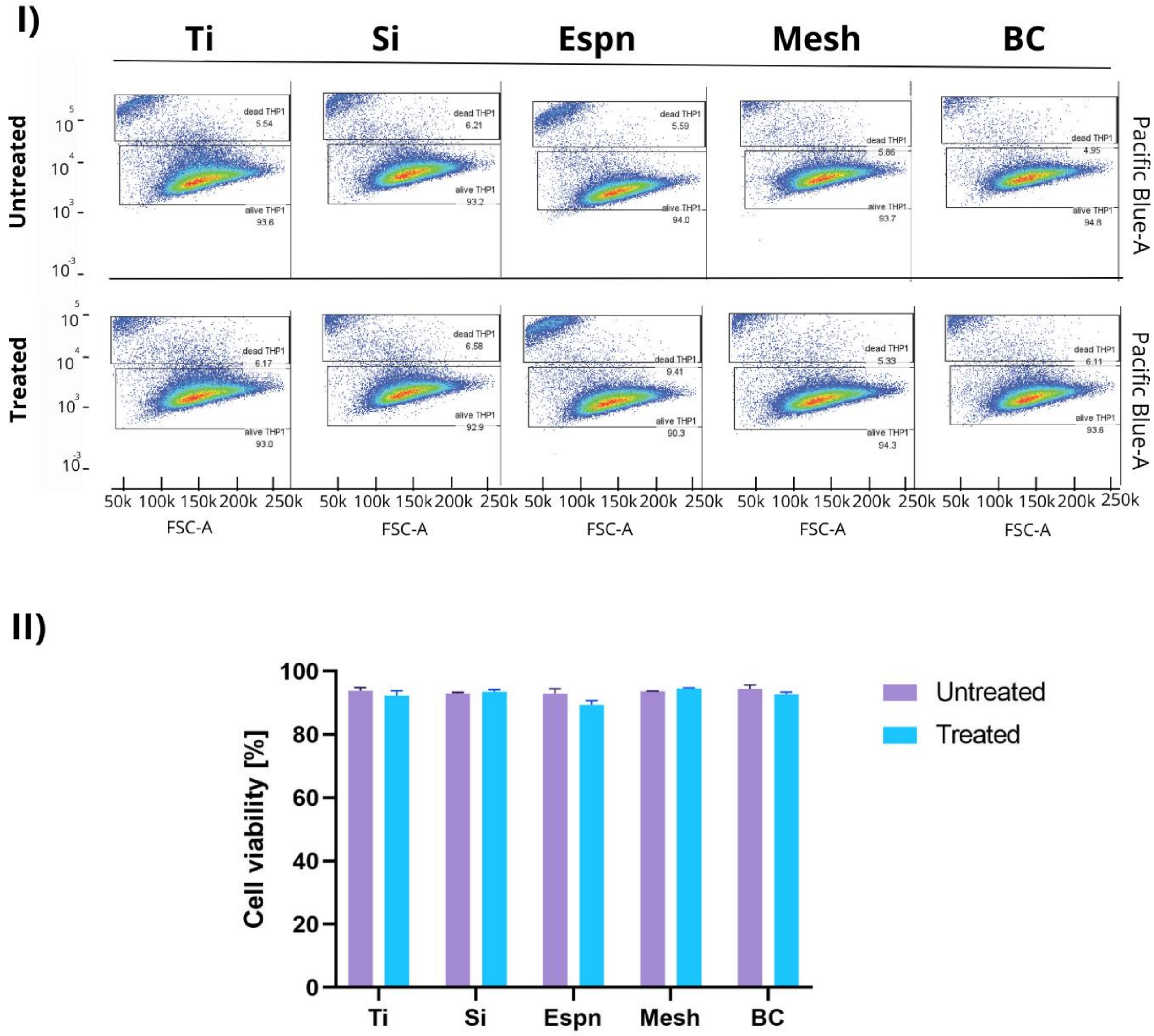

Supplementary Figure S1: Percentage of live THP-1 cells, defined as cell viability, when exposed to Titanium, Silicon, Electrospun PLGA, PGA mesh and Biocellulose treated with SET-M33 (15 µM). The percentage of viability is calculated over the total amount of cells per sample. Viability on treated samples is compared to viability of untreated controls. The data in the graph shows the percentage of viability as mean plus standard deviation (SD) among three measurements for each sample.

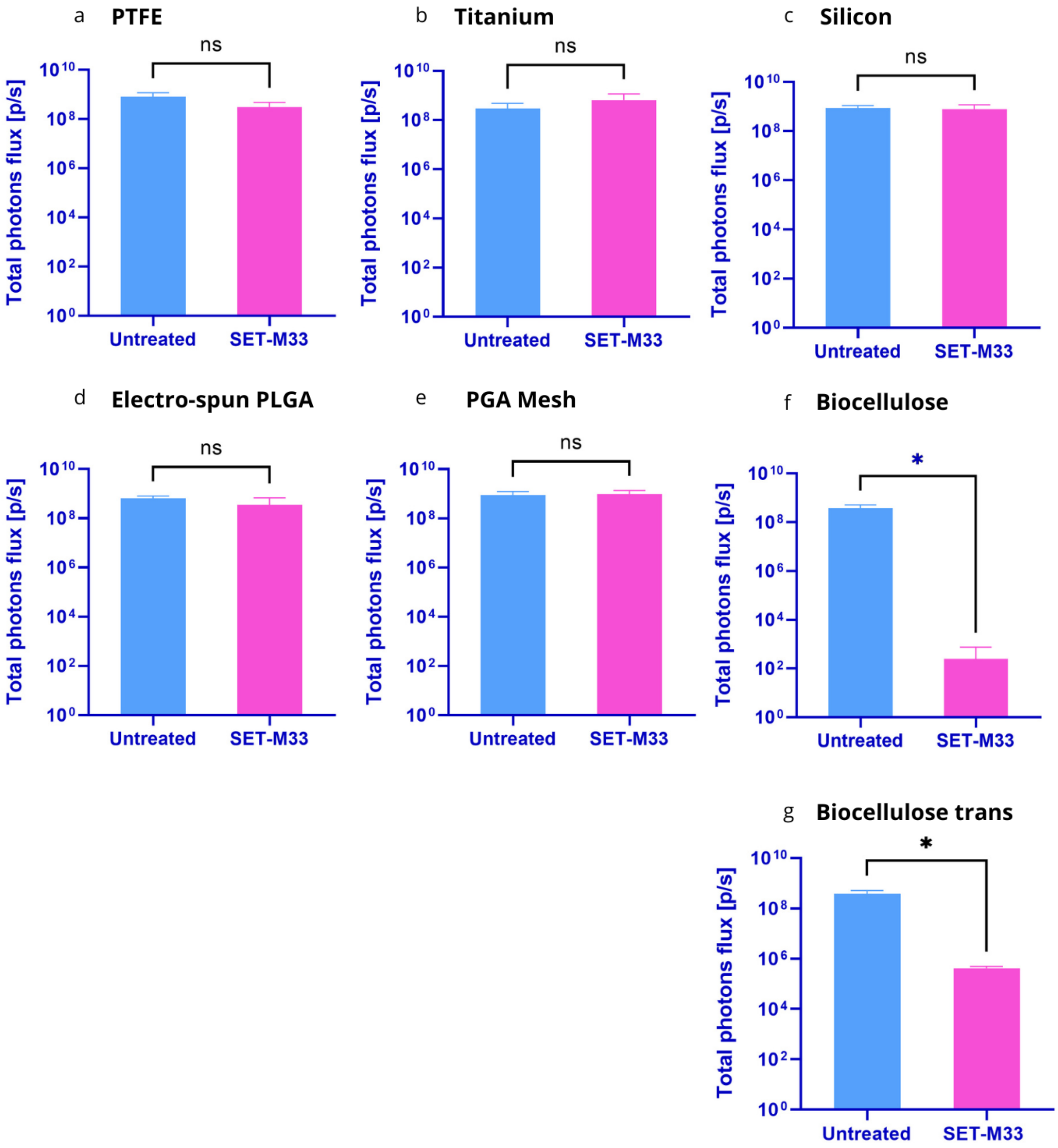

Supplementary Figure S2: Bioluminescence quantification via photons emission per ROI of PTFE (a), Ti (b), Si (c), Espn (d), Mesh (e), BC (f) and BC trans configuration treated with 15 μM SET-M33L compared to untreated controls. Bars indicate mean and standard deviation (SD) among four replicates for each sample. Each graph represents one of two independent experiments that produced consistent results. * Represents significant difference ($p < 0.05$) between the treated sample and the untreated control assessed by t-test.

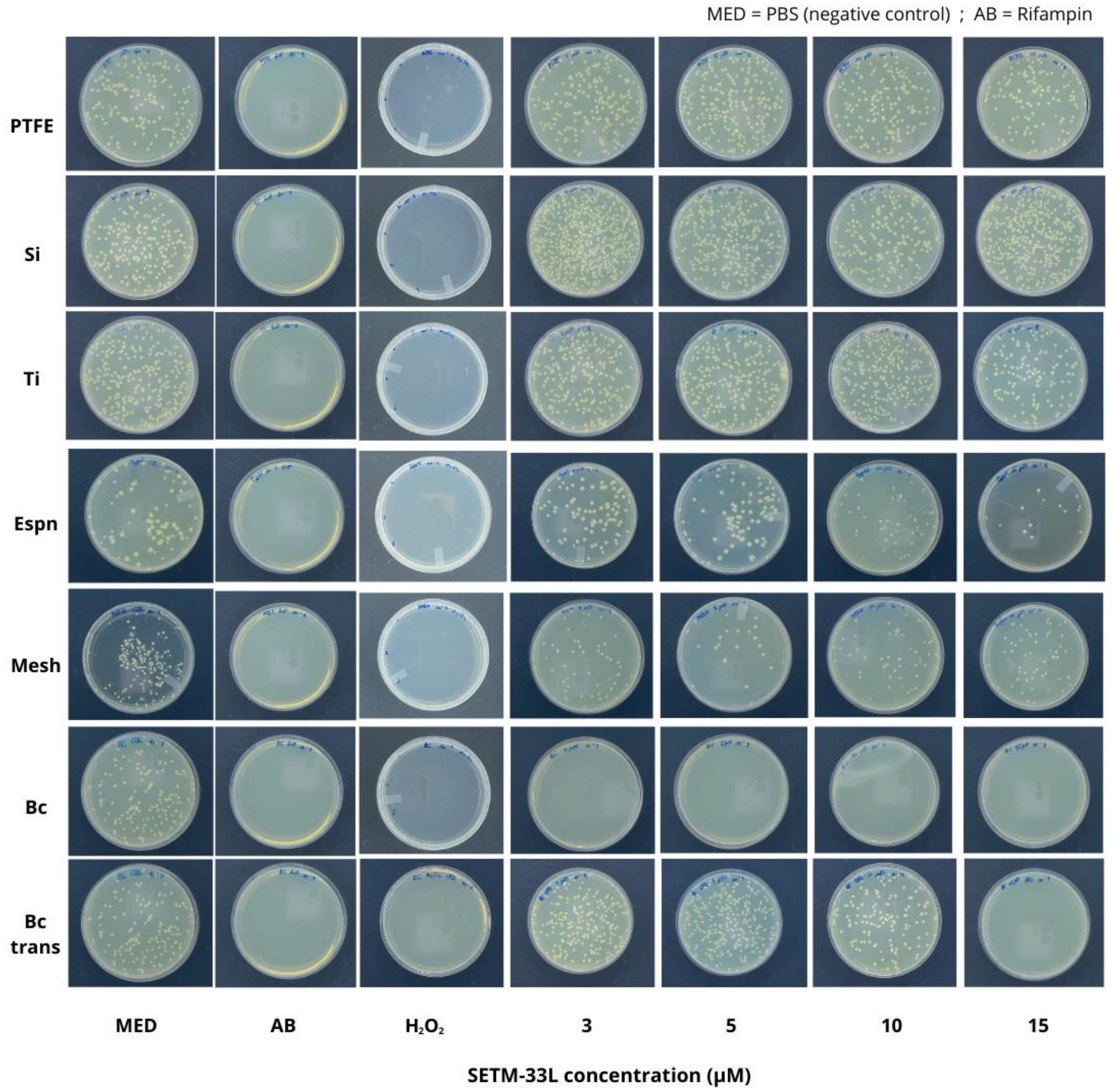

Supplementary Figure S3: Picture of agar plates for the different experimental conditions. All the plates shown in the figure refer to 10^{-4} dilution of the culture supernatant.

Supplementary tables

Supplementary Table S1: The table displays the difference in the Water Static Contact Angle (L: left, R: right) measured on the implant and envelope materials before and after treatment with antimicrobial peptide SET-M33L (15 µM). Values related to all the measurements are presented as mean ± SD among three replicates.

Implant Materials	Water Static Contact Angle (°)	
	Untreated	SET-M33 treated
**PTFE**	L: 107.35 ± 1.63<br>R: 109.62 ± 1.75	L: 40.65 ± 2.44<br>R: 45.17 ± 2.96
**Ti**	L: 78.50 ± 0.49<br>R: 74.80 ± 0.54	L: 18.83 ± 0.79<br>R: 16.67 ± 0.45
**Si**	L: 77.41 ± 0.08<br>R: 81.92 ± 0.17	L: 53.73 ± 2.48<br>R: 59.09 ± 2.56
**Envelope Materials**		
**BC**	L: 12.66 ± 0.60<br>R: 12.37 ±1.93	L: 12.66 ± 0.60<br>R: 12.37 ±1.93
**Espn**	L: 100.05 ± 0.49<br>R:102.23 ± 0.36	L: 83.36 ± 2.60<br>R:92.23 ± 3.01
**Mesh**	N.A	N.A

Supplementary Table S2: List of primers used to generate the final plasmid expressing CBG2 and miniSOG under the J23119 constitutive promoter.

Gene	Primer name	Primer sequence
JCrT1	pRNA Fow (JCrT1)	GTTAAGGCTGGCGGCGTTTAACCAGGCATCAAATAAAACGAAAGGCTCAGTCGAAAG
	pRNA Rev (JCrT1)	TGATGTTTCATACATACCTCCTTAGTCGGCGCTAGCATTATACCTAGGACTGAGCTAGC
	CBG2 Fow	TAATGCTAGCGCCGACTAAGGAGGTATGTATGAAACATCACCATCACCATCATGTTAAG
	CBG2 Rev	CGTTTTATTTGATGCCTGGTTAAACGCCGCCAGCCTTAAC
JmrT1	pRNA Fow (JmrT1)	ATAACCAGGCATCAAATAAAACGAAAGGCTCAGTCGAAAG
	pRNA Rev (JmrT1)	GTATGGGTACATACATACCTCCTTAGTCGGCGCTAGCATTATACCTAGGACTGAGCTAGC
	mSOG Fow 1	ATGTATGTACCCATACGATGTTCCAGATTACGCTGAGAAAAGTTTCGTGATAACTGATCC
	mSOG Fow 2	AATGCTAGCGCCGACTAAGGAGGTATGTATGTACCCATACGATGTTCCAGAT
	mSOG Rev	CTTTCGACTGAGCCTTTCGTTTTATTTGATGCCTGGTTATCCATCCAGCTGCACTCC
JmrT1 Kan	BackBone Fow	TTCTTCTGAGCGGGCTGTCAGACCAAGTTTACTCATATATACTTTAGATTGATTT
	BackBone Rev	ATCCATCTTGTTCAATCATACTCTTCCTTTTTCAATATTATTGAAGCATTTA
	Neo Fow	TAATATTGAAAAAGGAAGAGTATGATTGAACAAGATGGATTGCACGC
	Neo Rev	ATATATGAGTAAACTTGGTCTGACAGCCCGCTCAGAAGAACTCGTCAAGAAGGCG
JCmrT1 Kan	JmrT1 Fow BamHI	CTCTGGATCCTTGACAGCTAGCTCAGTCCTAGG
	JmrT1 Rev XbaI	TCAATCTAGAATGCATCGATGATAAGCTGTCAAACA
	JCrT1 Fow XbaI	GCATTCTAGATTGACAGCTAGCTCAGTCCTAGG
	JCrT1 Rev BamHI	TCAAGGATCCAGAGTTTGTAGAAACGCAAAAAGGC
